# Compassion Satisfaction and Fatigue in Bangladeshi Americans Providing Care to Aging Relatives

**DOI:** 10.1093/geroni/igaf122.3318

**Published:** 2025-12-31

**Authors:** Mohammad Hossain, Natalie Pope, Abigail Latimer

**Affiliations:** University of Kentucky, Lexington, Kentucky, United States; University of Kentucky, Lexington, Kentucky, United States; University of Kentucky, Lexington, Kentucky, United States

## Abstract

Caring for aging parents is deeply rooted in Bangladeshi culture. However, despite the significant growth of the Bangladeshi population in the US, there has been limited research on the well-being of Bangladeshi American family caregivers. This study examined compassion satisfaction (CS) and compassion fatigue (CF) among Bangladeshi Americans caring for aging relatives. We conducted a cross-sectional survey of 12 participants using the Professional Quality of Life (ProQOL) Scale, which includes a measure of CS and two measures of CF (burnout (BO) and secondary traumatic stress (STS)). The ProQOL is validated for use in family caregivers. The sample was equally distributed between both sexes, age (M = 41.44 ± 6.5) reporting moderate levels of CS (M = 38.33 ± 6.53), STS (M = 28.17 ± 9.30) and BO (M = 23.25 ± 5.42). A Pearson’s correlation analysis revealed a strong, positive, and significant correlation between caregivers’ birth order, r(12)= .74, p < .01) and number of siblings (r(12)= .61, p < .05) and CS. Those caregivers born later in their families and those with a higher number of siblings had more satisfaction with their caregiving experiences. There were no significant relationships found between age, birth order, number of siblings and CS and CF. While this sample is small, it is the first to explore caregiving experiences in Bangladeshi Americans, providing insight into an overlooked population. Future research may want to consider familial structures as protective factors to CF.

